# HIV-1 Nef Breaches Placental Barrier in Rat Model

**DOI:** 10.1371/journal.pone.0051518

**Published:** 2012-12-11

**Authors:** Poonam Singh, Saurabh Kumar Agnihotri, Mahesh Chandra Tewari, Sadan Kumar, Monika Sachdev, Raj Kamal Tripathi

**Affiliations:** 1 Toxicology Division, Central Drug Research Institute (Council of Scientific and Industrial Research), Lucknow, Uttar Pradesh, India; 2 Endocrinology Division, Central Drug Research Institute (Council of Scientific and Industrial Research), Lucknow, Uttar Pradesh, India; Lady Davis Institute for Medical Research, Canada

## Abstract

The vertical transmission of HIV-1 from the mother to fetus is known, but the molecular mechanism regulating this transmission is not fully characterized. The fetus is highly protected by the placenta, which does not permit microbial pathogens to cross the placental barrier. In the present study, a rat model was established to observe the effect of HIV-1 protein Nef on placental barrier. Evans blue dye was used to assay permeability of placental barrier and fourteen day pregnant Sprague Dawley rats were injected intravenously with 2% Evans blue dye along with various concentrations of recombinant Nef. After an hour, animals were sacrificed and dye migration was observed through the assimilation of peripheral blood into fetus. Interestingly, traces of recombinant Nef protein were detected in the embryo as well as amniotic fluid and amniotic membrane along with placenta and uterus. Our study indicates that recombinant HIV-1-Nef protein breaches the placental barrier and allows the migration of Evans blue dye to the growing fetus. Further the concentration of Nef protein in blood is directly proportional to the intensity of dye migration and to the amount of Nef protein detected in uterus, placenta, amniotic membrane, amniotic fluid and embryo. Based on this study, it can be concluded that the HIV-1 Nef protein has a direct effect on breaching of the placental barrier in the model we have established in this study. Our observations will be helpful to understand the molecular mechanisms related to this breach of placental barrier by Nef in humans and may be helpful to identify specific Nef inhibitors.

## Introduction

Blood brain barrier (BBB) and placental barrier (PB) are the two protective barriers that protect brain and embryo respectively, from migration of proteins and toxic substances from peripheral blood. At the same time these barriers allow the migration of essential nutrients that are useful for the development during organogenesis. BBB protective mechanism persists constitutively through out the life where as the PB is activated after embryo implantation. These barriers also have the property to act like an insulator to protect the brain and placenta from infectious diseases. However few virus and bacteria are known to breach these barriers [1], [2].

The mechanism of BBB has been broadly studied [3], [4] but the precise mechanism by which some substances breach this barrier has not been characterized. The breach of PB may allow in-utero transmission of infections. For e.g., HIV transmission from mother to child is reported in the range of 25 to 35% [5]. Intrauterine transmission of HIV-1 is presumably due to multiple factors like low CD4 counts, high viral load, neutralizing antibody, other maternal immune factors, genetic factors, and any corrosive morphological changes in intact placenta [6], [7] but the precise mechanism of utero transmission has not yet been completely understood.

Placental membrane, which exists at uterine-placental interface, separates the fetal blood from the maternal blood. It consists of fetal vascular endothelium, connective tissues, chorionic villi, and trophoblasts, where anchoring villi attaches the placenta to the uterine. The chorionic villous contains macrophages (Hofbauer cells) and fetal vessels. The trophoblast and terminal villi have CD4 receptors [8] and stay in direct contact with the maternal blood.

HIV-1 genomic material has been detected in Hafbauer cells as well as in trophoblast cells [9], [10], [11]. At the same time HIV-1 also has been identified on both the maternal and the fetal parts of the placenta and shown to be replicated in the placenta. The virus may cross the trophoblastic barrier by endocytosis, or by an injured villous surface [12]. There are reports that this transmission requires cell to cell contact and it has been speculated that T cell to placenta contact could be responsible for infection of trophoblast by HIV-1 and this was inhibited by anti-LFA-1 antibodies [13]. Perinatal transmission of HIV-1 has been characterized by selection of specific genotype variants in the infected infants that escapes mother’s immune system [14]. It is also known that concomitant placental infection may increase the risk of placental transmission of HIV-1 to the fetus [12].

Microbial and viral proteins have been reported play an important role in intra uterine transfer of infections. The bacterial proteins InlA and InlB from Listeria monocytogenes enables bacteria to cross placental barrier and causes fetoplacental listeriosis [15]. The HIV-1 proteins; vif, vip, vpu, and Nef have been reported to be associated with intrauterine transmission of infection from mother to child [16, 17, 18 and 19]. Interestingly, the protein Nef has been shown to be a pathogenic protein which has multiple functions that accelerates viral pathogenesis and is suspected to be responsible for HIV-1 vertical transmission. It has been reported that Nef open reading frame was maintained in both mother and infant with a frequency of 86.2%, with the functional domains important of the activity of the protein conserved in both mother and the infant to whom it was transmitted [19]. Exposure of Nef to astrocytes show expression of proinflammatory cytokines including caspase 3, complement factor 3(C3) and the production of total nitrate and reactive oxygen species. The inflammatory cytokines, nitrates and reactive oxygen species have been associated with break of BBB [20]. However, none of the studies have clearly demonstrated the role of Nef in breaching of placental barrier. Moreover, the presence of alternate chemokine receptors, CXCR4 expressed on trophoblast may also play an important role in utero transmission [21]. LIF, a member of the IL-6 cytokine family, is up-regulated in the placenta of non transmitting women compared to transmitters [22].

In the present study, we have established a rodent model for demonstrating the breach of placental barrier by the HIV-1 protein. We have shown direct effect of Nef in breaching placental barrier, where recombinant Nef protein along with Evans blue dye was injected intravenously in fourteen day pregnant Sprague Dawley rat. The migration of the Evans blue dye from pheripheral blood to growing embryo and the breach of the placental barrier was studied in systemic manner. The presence of peripheral Nef protein in placenta, amniotic membrane, amniotic fluid and embryo was confirmed using Nef specific antibodies in ELISA as well as immuno-fluorescent assays. These results provide direct evidence about a functional role of Nef protein in the breach of placental barrier of the rodent model and the studies would be helpful to correlate the mechanism in other higher mammals.

## Results

### Effect of Nef on Migration of Evans Blue Dye Through the Placenta

**Figure 1:**
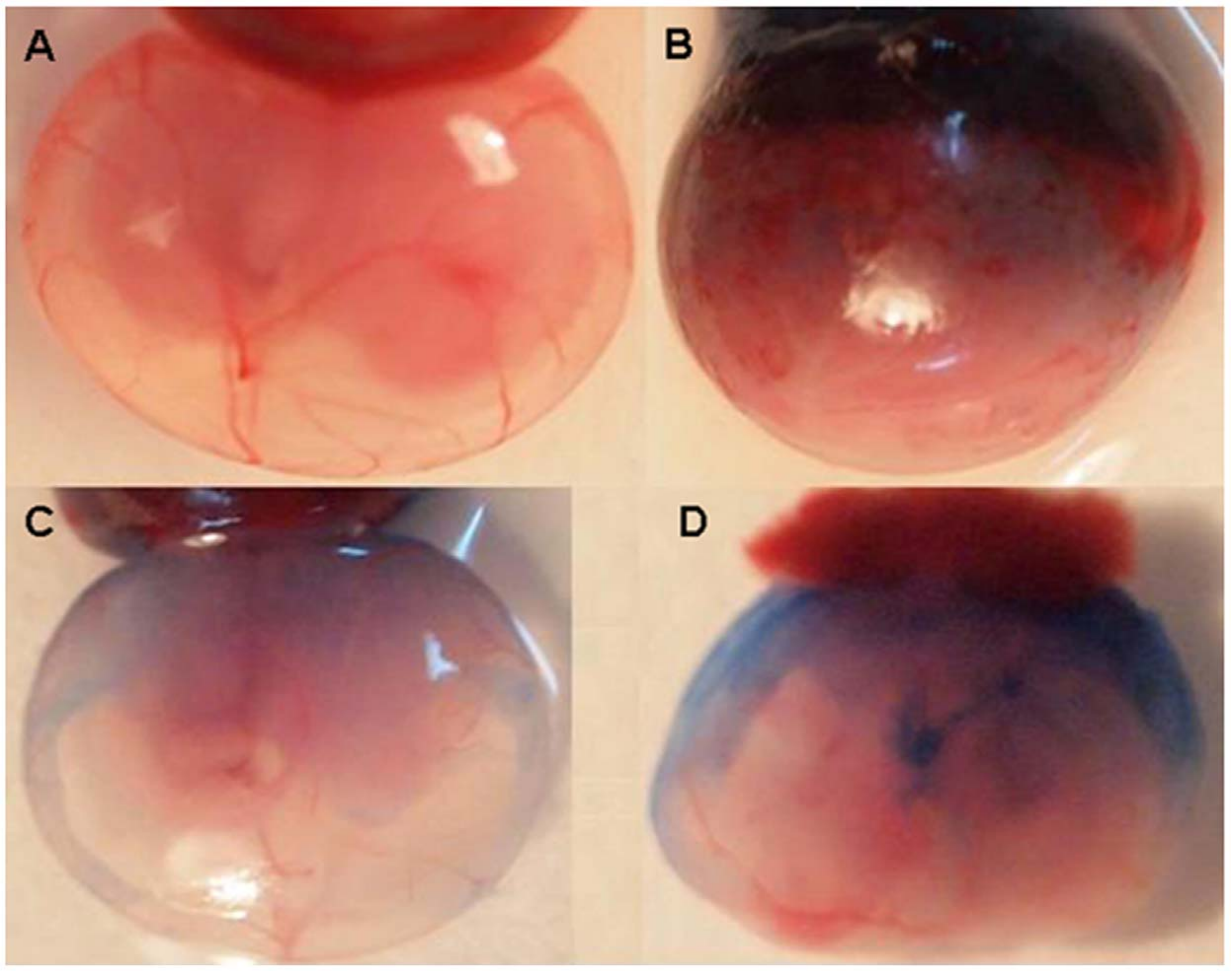
Evans blue dye migration in rat whole embryo after an hour of injecting recombinant Nef at different concentration. A: Without injection, B: Only Dye, C: Dye+250 µg Nef, D: Dye +500 µg Nef.

**Figure 2:**
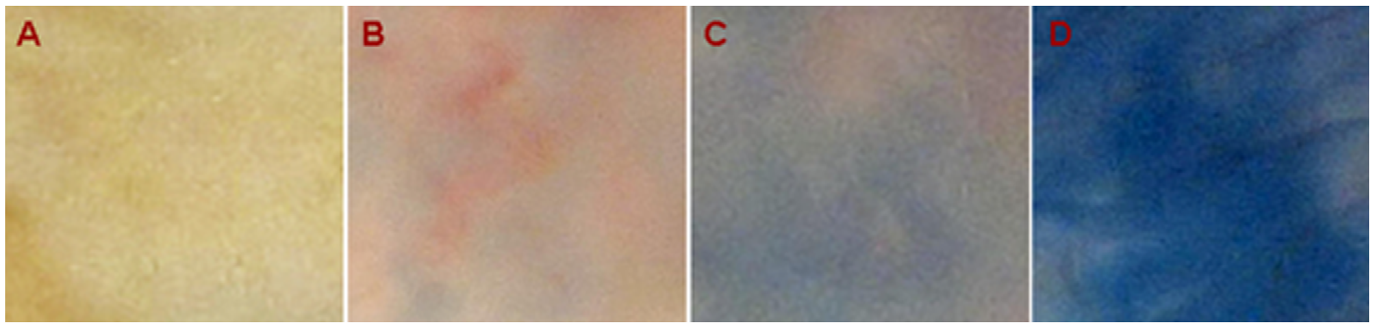
Entrenched Evans blue dye in the amniotic membrane of rat fetus, after an hour of intra-venous injection with different concentrations of recombinant Nef. A: Without injection, B: Only Dye, C: Dye+250 µg Nef, D: Dye+500 µg Nef.

An earlier report showing the effect of Nef on blood brain barrier [23] directed our approach to study the effect of Nef on Placental barrier. One of the possible mechanism for vertical transmission of HIV-1 from mother to child is breach of placental barrier, in the present study various concentrations of the recombinant Nef protein in the range of 0–500 µg (Table S1) and 500 µg of ASK-1 host protein was injected intravenously in fourteen days pregnant rats along with 2% of Evans blue dye. Animals were sacrificed after an hour of the injection and checked for the breach of placenta. In a group of rats, where only the dye was injected without Nef; it was observed to concentrate at the placental junction and the migration of dye was not visible in the amniotic membrane. Interestingly, the rats which were injected with Nef showed migration of Evans blue dye throughout the amniotic membrane and the intensity of dye decreased at the junction (Figure 1). Further when a piece of amniotic membrane was taken from the non-injected as well as injected animals, a clear visual effect of the increased blue color due to dye migration was observed (Figure 2). Amniotic membrane of the animal injected with 500 µg of Nef showed a strong blue color, while a slightly lesser intensity of blue color was observed in the animal injected with 250 µg of Nef. A very light blue splash was observed in the amniotic membrane in the animal where only the dye was injected without Nef. In the non-injected animal, the original pink color of the amniotic membrane was visible.

**Figure 3:**
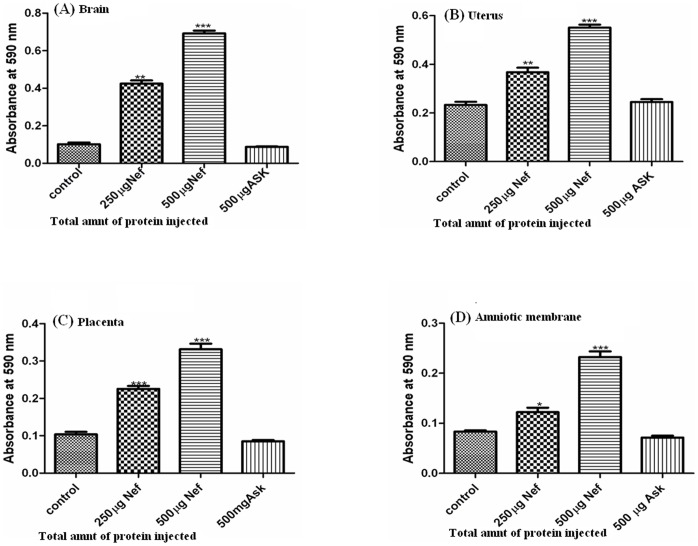
Quantification of Evans blue dye (OD at 590) present (within an hour) in brain and different fetal tissue isolates from 14 day pregnant Sprague Dawley rats injected intravenously without and with recombinant Nef and ASK-1 protein. (A) Brain (B) Uterus (C) Placenta (D) Amniotic membrane. Three different bars in each set represent 0, 250 and 500 µg of recombinant Nef and 500 µg of ASK-1 injected intravenously along with Evans blue dye in the experimental animals. As data represent ±SEM of 3 separate experiments in duplicate and changes were considered as significant at *p≤0.05, **p≤0.01 and ***p≤0.001.

The migration of the dye color was quantified by measuring the optical density at 590 nM in the lysates of different fetal tissues. In the animals injected 250 and 500 µg of Nef, the tissue lysates showed almost two to four fold increase in the dye migration, respectively as compared to the animals in which the ASK-1 protein and dye was injected without Nef (Figure 3B, C and D). The threshold level of Nef required to breach the placental barrier was analyzed with varying levels of Nef injection (25–500 µgs of Nef protein corresponding to 10–865 ng/ml of plasma Nef; Table S1). An injection of 200 µg of Nef analogous to ∼500 ng/ml Nef in the plasma was observed to result in partial breach of placental barrier and the blood brain barrier. Migration of the dye in the presence of Nef was also observed in the brain as compared to ASK-1 protein and only dye injected rats indicating that the constitutive blood brain barrier is breached (Figure 3A). The migration of dye was also observed in the amniotic fluid of Nef injected rats (Figure 1). Our results clearly indicate that Nef is involved in permeabilization of both blood brain barrier as well as placental barrier suggesting the direct role of Nef in the process of breaching the barriers.

### Migration of Recombinant Nef from Peripheral Blood to Fetal Tissues

**Figure 4:**
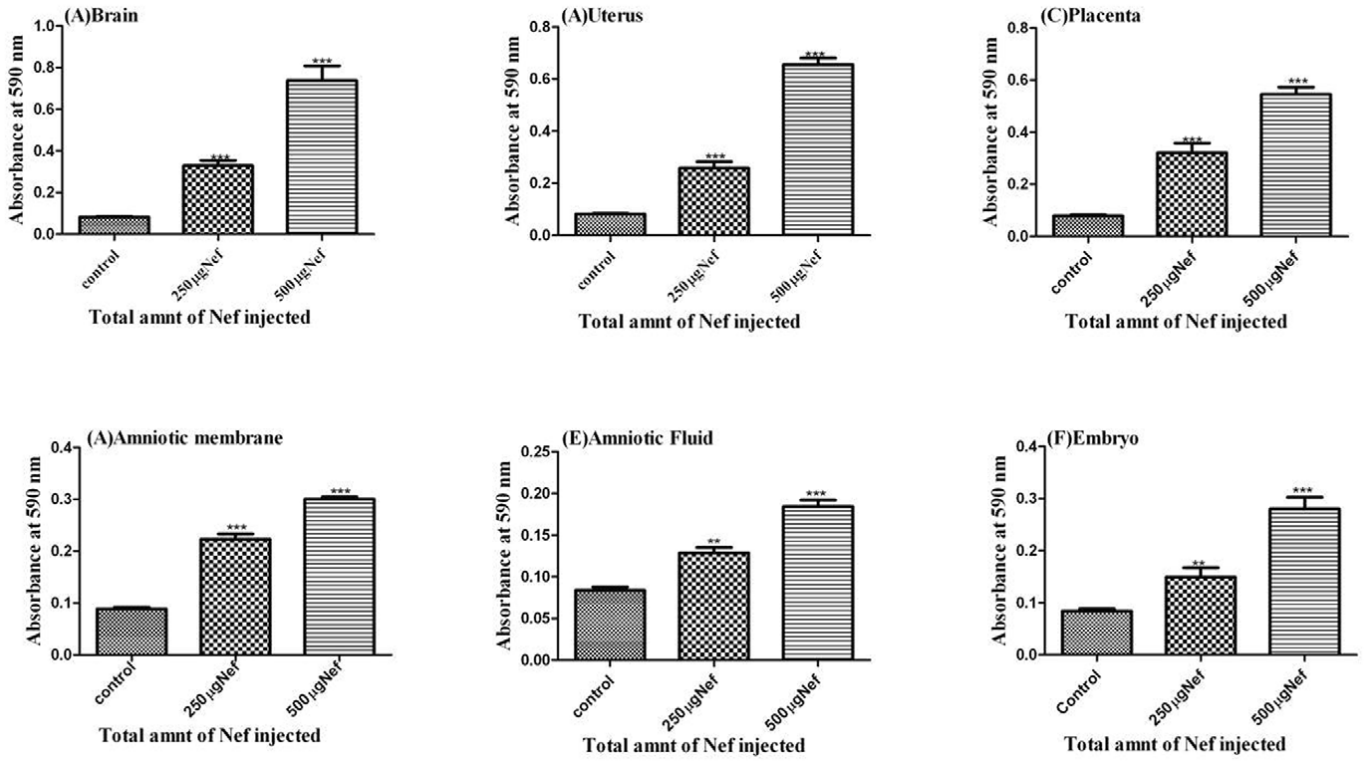
Detection of intravenous recombinant Nef protein migrated (within an hour) in brain and different fetal tissue isolates from 14 day pregnant Sprague Dawley rats injected intravenously without and with recombinant Nef protein. (A) Brain (B) Uterus, (C) Placenta, (D) Amniotic membrane, (E) Amniotic fluid and (F) fetus after breaching the placental barrier. Three different bars in each set represents 0 µg, 250 µg and 500 µg of recombinant Nef injected intravenously along with Evans Blue dye in these animals As data represent ±SEM of 3 separate experiments in duplicate and changes were considered as significant at *p≤0.05, **p≤0.01 and ***p≤0.001.

**Figure 5:**
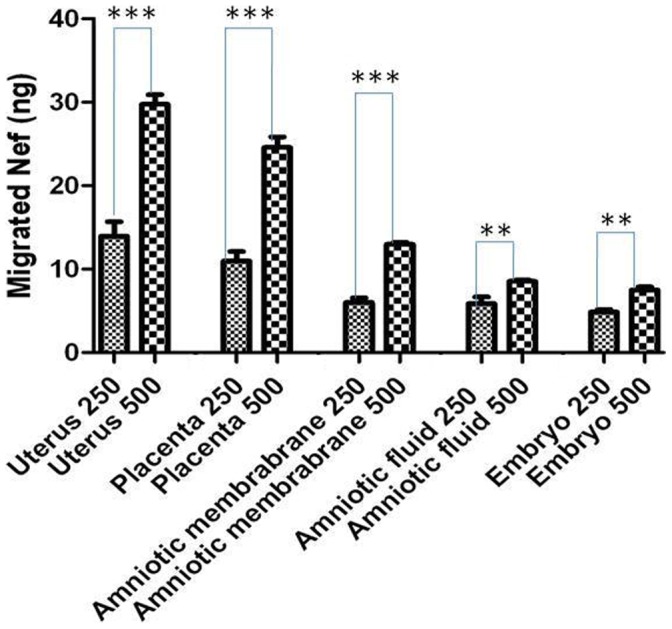
Quantification of migrated recombinant Nef (within an hour) in fetal tissues. Uterus, Amniotic membrane, Amniotic fluid and Embryo of female rats injected with 250 µg and 500 µg of recombinant Nef protein intravenously. As data represent ±SEM of 3 separate experiments in duplicate and changes were considered as significant at *p≤0.05, **p≤0.01 and ***p≤0.001.

The migration of dye from placenta to fetal tissues indicates the mixing of peripheral blood in fetus. The outflow of peripheral blood in fetus also involves the migration of proteins and other components which would be exclusively present in blood and usually do not cross the placental barrier. Therefore once the placental barrier breached, the peripheral recombinant Nef protein was identified in different fetal tissues. The protein lysates of uterus, placenta, amniotic membrane, amniotic fluid and embryo were analyzed for the existence of peripheral recombinant Nef protein through ELISA using Nef specific antibody (Figure 4). Our analysis showed the existence of recombinant Nef in all the fetal tissues in animals injected with Nef as compared to the control animals which were not injected with Nef, but only with the dye. The concentration of Nef (assessed by ELISA) in the fetal tissues was increased from two fold to four fold, when the dose of injected recombinant Nef was increased from 250 µg to 500 µg (Figure 4). Figure S3 displays the standard graph for assessment of Nef concentration of unknown samples. The average quantity of migrated Nef was calculated in all the fetal tissues of animals injected with a range of Nef (Table 1 and Table S1). After the breach of PB; in uterus, it was observed to be 13.9 ng and 29.6 ng; in placenta 10.9 ng and 24.5 ng; in amniotic membrane 5.6 ng and 12.8 ng; in amniotic fluid 6.0 ng and 8.5 ng; and in the embryo 3.6 ng and 7.4 ng, respectively in animals injected with 250 µg and 500 µg of Nef (Figure 5). Our study has clearly illustrated that Nef breaches the placental barrier and allows mixing of peripheral blood in fetal tissues, at the same time the amount of Nef protein in blood is critical (Table S1) to decide the extent of breach of placental barrier. Studies from different groups also showed that Nef breaks blood brain barrier in mice and rats [24], [25], [26].

**Table 1:** Amount of Nef detected in plasma and migrated in fetal organs after the intra venous injection of HIV-1 Nef protein.

Nef Injected (µg)	Nef conc. in Plasma(ng/ml)	Nef Migrated in Different Fetal Organs (ng/ml)				
		Uterus	Placenta	Amniotic membrane	Amniotic fluid	Embryo
**250**	584	13.96	10.97	6.014	5.86	4.88
**500**	865	29.75	24.58	12.99	8.53	7.50

We also analyzed the effect of intravenous Nef on blood brain barrier in pregnant rats. The presence of peripheral recombinant Nef protein in brain tissue lysates (Figure 4A) was clearly noticed. Even though the quantity of the migrated Nef was quiet low in comparison to the quantity of Nef injected (Figure 4 and 5) we could still observe its presence in the brain.

### Localization of Recombinant Nef Protein in Fetal Tissues

**Figure 6:**
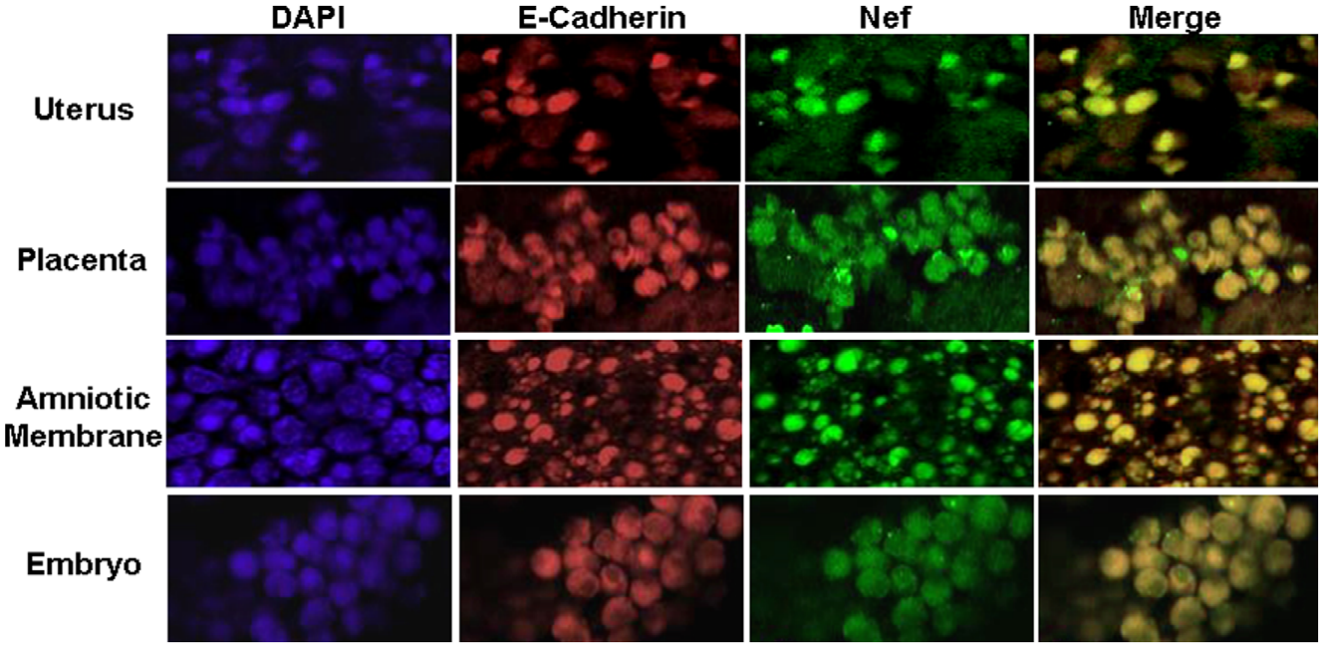
Localization of recombinant Nef protein (green) and E-cadherin (red) in different fetal tissues through immuno-fluorescence staining. The panel consist of set of figures; uterus, placenta, amniotic membrane and embryo. Blue colour shows the nuclear staining with DAPI whereas overlapping images are merged for Nef and E-cadherin localization. The fetal tissues were isolated from rats after an hour of intra-venous injection with 500 µg of recombinant Nef protein.

The migration of recombinant Nef protein from peripheral blood to the fetal tissues as well as in brain has been demonstrated in our experiments mentioned above. In order to confirm the migration of Nef traces in the fetal tissues, these tissues were obtained and fixed immediately from the animal set injected with 500 µg of Nef protein. Paraffin-embedded tissue sections were used to track recombinant Nef protein through immuno-flourescence (Figure 6). Cell-specific marker E-cadherin was also localized simultaneously in these tissue sections. Nef could be clearly localized in the tissues, confirming the presence of entrenched Nef in the uterus wall, placenta as well as amniotic membrane and the growing embryo. Nef staining cells were found to be overlapping with E-cadherin, demonstrating the intracellular localization of Nef. At the same time in a few marginal areas of placenta, Nef was also observed to be extracellular. When these sections were probed with only secondary antibodies, no signal was observed (Figure S1). This indicates the specificity of the antibody we have used in this study against Nef. In the animals where the dye was injected alone without any recombinant Nef (i.e. negative control) no signal was observed (Figure S2), which further confirms the specificity of Nef. These results suggest that recombinant Nef is involved in breaching of placental barrier and migration to the growing embryo; at the same time may be liable for the intra uterine transfer of other components present in peripheral blood.

## Discussion

As per the available literature, HIV-1 Nef protein has been shown to be a pathogenic protein and have multi functions that are responsible for viral pathogenesis [27] and it is also suspected to play a role in the vertical transmission of HIV-1 from mother to fetus [19]. Usually the fetus is highly protected by placenta which has a barrier that does not permit the migration of microbes. However, in some cases, this barrier is breached. How this placental barrier is breached and how HIV does cross this barrier is a still stimulating question to be answered. In order to gain an understanding about this mechanism, for the first time, we developed a rat model closely related to human, for studying the effect of HIV-1 protein Nef on placental barrier.

Our results show that HIV-1 accessory protein Nef breaches placental barrier in 14 day rat embryo. The evidences we present in our study are in terms of migration of Evans blue dye in Nef injected rats across the placental barrier and localization of the protein and the dye in uterus, placenta, amniotic membrane as well as amniotic fluid in the rats treated with Nef, while no such migration is seen in the negative control sets (Figure 1). Breaching the placental barrier and blood brain barrier causes the mixing of peripheral blood to fetus and brain respectively. It is known that blood borne maternal pathogens that arrive at the utero-placental circulation and intervillous spaces may reach the fetus through the villous capillaries. The migration of dye (as observed in the present study) in the peripheral blood is possibly, due to the rupture of tight junctions of endothelial cells of placenta and villous capillaries. Based on our observations, it can be assumed that Nef is responsible for breaking of the tight junctions of endothelial cells. Studies involving Japanese encephalitis virus have demonstrated that macrophage-derived neutrophil chemotactic factor (MDF) and Dengue Virus mediated cytotoxic factor (CF) are responsible for migration of plasma protein bound dye from peripheral blood to brain after breaching of blood brain barrier [24], [25]. In the present study, the free migration of dye in presence of Nef from uterus to embryo and from blood to brain indicated that Nef is indeed responsible for the breach of placental barrier as well as increased permeability of placenta and the blood brain barrier.

Further, we also observed that the recombinant HIV Nef protein injected intravenously in 14 day pregnant rats, showed up its in traces in uterus, placenta, amniotic membrane, amniotic fluid and embryo. This clearly demonstrates that Nef increases the placental permeability possibly by breaking the tight junctions of endothelial cells of villous capillaries and facilitates the migration of the protein to the embryo.

It has been earlier speculated that pathological conditions of placenta of HIV-1 mothers for e.g. chorioamnionitis and cytotrophoblastic hyperplasia may be responsible for breaching of placental barrier. However, there is no relationship between the incidences of placenta pathological lesions with the HIV-1 status of new born [28]. Further, it has been observed that the incidences of chorioamniotitis increase in HIV-1 infected women [29]; therefore there must be other mechanism responsible for intra-uterine transmission of HIV and our study indicates that Nef protein is possibly one of the factors responsible for this transmission.

HIV-1 Nef has been earlier clearly demonstrated to be a pathogenic protein and it shows AIDS like pathogenesis in both *C.elegans* [30] and mice [31]. Nef interacts with more than thirty different host proteins regulating immune evasion, apoptosis, signaling, activation of T cell [32], [33]. Therefore, it could be possible that Nef may interact with proteins present in endothelium and result in permeabilization of the tight junctions of connective tissues. Moreover, Nef present in uterus, placenta, amniotic membrane, amniotic fluid and embryo may be interacting with other fetal proteins, thus enhancing the adverse effect on placental barrier. There is evidence that Nef breaks blood brain barrier in mice [23] and our study also proves the same in the rat model, suggesting that Nef affects the constitutive barrier in both the rodents. Moreover our study has clearly illustrated for the first time, the breach of placental barrier through Nef.

It is know that cytokines play vital role in pregnancy through a response called Th2 type immune response. Cytokine and chemokines expressed at fetus-placental junction could serve as markers for understanding the intrauterine transfer of HIV-1. Placenta of non transmitting mother sustain strong TH2 cytokine environment, where as mother from transmitting HIV-1 demonstrate strong Th1 cytokine environment [34]. LIF, a member of the IL-6 cytokine family, is upregulated in the placenta of non-transmitting women compared to HIV-1 transmitters [22]. It has been seen that Nef induces pro-inflammatory cytokines, production of nitrate and reactive oxygen species in astrocytes which could be responsible for the breach of BBB in mice [20], [23]. Nef protein also can induce BBB disruption in rat, presumably via MMP induction [26] but so far no studies could demonstrate the clear role of Nef in breaching the PB. However, indirect evidence has been shown that the inflammatory cytokines expressed in placenta favors the HIV-1 transmission to fetus. The treatment with AZT to HIV-1 infected pregnant women down- regulated TNF-α mRNA expression in placental cells [35]. Down-regulation of TNF-α mRNA could represent a mechanism through which AZT can decrease the risk of HIV-1 in utero transmission, in addition to its direct effect on HIV-1 replication. Since the similar signaling molecules do exists in placenta, it can be hypothesized that a similar mechanism could be inducing inflammatory cytokines which ultimately result in breaching of PB. Through the present study, an excellent model has been established to study the molecular mechanisms regulating the signaling pathway and upregulation of inflammatory cytokines for the breach of placental barrier through Nef. We are presently characterizing the role of Nef in permeabilization of the endothelial cells of placenta.

High HIV-1 load has been assumed to be a strong reason for mother to child transmission of HIV-1 [6], [36]. Similarly HBV levels in pregnant woman serum and fetal blood also have been correlated [37]. There is no threshold values of viral load or Nef protein have been identified to distinguish between transmitting and non-transmitting mothers [38]. Tat and Nef are the viral proteins expressed early after HIV-1 infection even before the formation of provirus [39]. Based on the results of our study, it could be speculated that a threshold concentration of Nef in serum (∼500 ng/ml Nef in the Rat model) is needed to break the placental barrier. The quantitative effect of Nef on placental barrier could not be studied in transgenic Nef mice as the concentration of Nef could not be regulated specifically [40], whereas the present study has the potential to study the quantitative effect of Nef *in vivo* (Figure 5).

Until now there is no established model of HIV-1 intrauterine transmission, which makes the understanding really difficult that how exactly this transmission occurs. Our study has opened a new research approach to comprehend the molecular mechanism of intrauterine transmission of HIV-1 through Nef protein in terms of a rodent model. We have demonstrated that HIV protein Nef mediate increased permeability of placental barrier in pregnant rats. Even though the dosage given to these animals was acute, still the apparent breach of placental barrier noticed in the rats clearly indicates a role for Nef in increasing the placental permeability and causing its breach. Even though most women with HIV do not show signs of leakage of the placental barrier, about 20–30% cases involving mother to child transmission of HIV has been reported. It is indeed persuasive to speculate based on the results of our study that this may be due to the breach of placental barrier caused by an increased viral load or enhanced expression of Nef or both. We have clearly demonstrated a threshold concentration of Nef needed to breach the placental barrier in rats, which can be considered acute. However, the threshold levels required for the placental breach may be different in the infected pregnant women and the results of the present study cannot be directly correlated with humans. An analysis of serum level of Nef in women and correlating it with the breach of placental barrier should reveal the role of Nef in mother to child transmission of HIV. The rodent model established in the present study can certainly be used to understand the molecular mechanisms involved in placental breach through Nef and identification of placental proteins interacting with Nef. This model also can be used to identify the novel Nef inhibitors subjected to seize the breach of placental barrier.

## Materials and Methods

### Expression and Purification of Recombinant HIV-1 Nef Protein

The HIV-1 Nef and human ASK-1 gene were cloned in T7 expression vector Pet 28a that has His6-tag at C-terminal. The recombinant Nef protein was expressed in C-41 bacterial host [41]. Briefly for both the proteins, 5 ml overnight bacterial culture was used to inoculate 1l ml of Luria-Bertani broth media containing 0.25 µg/ml kanamycin. Cultures were grown at 37°C until the OD reached 0.6, followed by induction for 18 hr with 0.8 mM IPTG. Then the culture was harvested and the pellets were re-suspended in binding buffer containing 25 mM Tris [pH 8.8], 50 mM NaCl as well as protease inhibitor PMSF. The resuspended pellet in buffer was sonicated for 20 minutes at amplitude 25 (with 10 sec on and 10 sec 0ff) and then centrifuged at 12500 rpm at 8°C for 30 min, and supernatant was collected. The soluble protein present in supernatant was purified by Ni-affinity chromatography with buffers containing 50 mM Tris (pH 8.8) and 25 mM NaCl, with increasing concentration of immidazole @ 25 mM, 50 mM, 75 mM and 300 mM in wash # 1, wash # 2, wash # 3 and in elution buffer respectively. The eluted protein from Ni-NTA column was concentrated further through concentrators (Millipore) for reducing the volume to 2–3 ml and then it was additionally purified by size exclusion chromatography. Purity and integrity of Nef and ASK-1 was confirmed through SDS/PAGE and western blot.

### Confirmation of Pregnancy in Sprague Dawley Rats

Mature females weighing around 160–180 gms and males weighing around 180–200 gms were placed together in the same cage for mating in the afternoon (1600–1800 hrs) in a ratio of 3∶1. Males copulate with females at proestrus around the midpoint of the dark cycle. Successful mating was confirmed by the presence of a vaginal plug in the females in the morning following copulation, which was considered day 1 of pregnancy. Vaginal swab was also collected gently and smear was prepared on a glass slide to examine the presence of sperms under the light microscope to confirm the natural mating as well as established pregnancy.

### Assay of Blood Brain Barrier and Placental Barrier Permeability

The Evans blue dye was used to assay permeability of blood brain barrier of fourteen day pregnant rats. Twenty pregnant Sprague Dawley rats were taken and divided them in to five groups as control (without injection), 0, 250 and 500 µg of recombinant Nef and 500 µg of ASK-1 in each group of four animals. Lower amount of Nef injections were also checked before to have an idea about the threshold value of Nef injection needed to breach the placental barrier (Table S1). The 2% Evans Blue dye was dissolved in normal saline of 0.85% sodium chloride and 500 µl of dye containing recombinant Nef at concentration of 0, 250 and 500 µg and ASK-1 500 µg were injected intravenously in the tail vein of each group of animals. After 1 hour all the rats including the un-injected ones were anaesthetized and dissected to take out complete uterus and the brain carefully. Further fetal tissues; uterus, placenta, amniotic membrane and embryo were separated cautiously and collected in PBS to measure the weight of these organs immediately. Each fetal tissue was homogenized in PBS (pH7.4) adjusting the tissue weight in mg/ml concentration. The homogenized tissue centrifuged at 10000 rpm at 4°C for 15 minutes and clear supernatant was collected. Now the absorbance (OD) was determined from the supernatant at 590 nm, which was calculated per mg of tissue weight for the quantitative analysis of Evans blue dye. The absorbance of Evans blue dye was measured at OD 590 from the brain as well as fetal tissues involved in blood-brain barrier and placental barrier respectively. Absorbance was taken as an average of four animals and the actual absorbance was calculated after normalizing the background values from the un-injected control set of tissues.

### ELISA

The tissue lysates of brain, uterus, placenta, amniotic membrane as well as amniotic fluid were homogenized in PBS containing 1% (w/v) Triton x-100 from the different set of rats. The homogenate was centrifuged at 10000 rpm for 15 min and clear supernatant was collected. Estimation of protein concentration for the lysate was done through Bradford method (Sigma). 96 well plates (Greiner) were coated with 50 µg of total protein from all the lysate samples in replicates and incubated at 37°C for 2 hrs. The supernatant was discarded and the wells were blocked for 2 hrs with 5% BSA in PBST and subsequently incubated with anti-Nef antibody (Santa-Cruze, USA) at 1∶500 dilution in 2% BSA for 2 hrs. The supernatant was discarded and five washes were done with PBST for 5 minutes each. These wells were incubated further with HRP-conjugated anti-donkey goat antibody (Santa Cruz) at a dilution of 1∶10,000 for 2 hrs, followed by five more washes with PBST for 5 minutes each and developed with TMB substrate (Pierce). The optical density was measured at 450 nm.

Simultaneously ELISA was done with the known quantities of recombinant Nef in the range of 0.25 to 25 ng to make standard graph (Figure S3). A standard graph was used to calculate the amount of recombinant Nef protein in the experimental samples.

### Immuno-florescence

The fetal tissues; uterus, placenta, amniotic membrane and embryo were separated from the rats and fixed in 4% para-formaldehyde for 10–15 hrs followed by dehydration with 70%, 80% and 90% isopropanol sequentially for 1 hr each and then incubated in 100% isopropanol for 1 hr, during which period the isopropanol was changed thrice. Subsequently the tissues were dehydrated in xylene for half an hour thrice and the blocks were prepared by paraffinising them in melted wax for 4–10 hrs. Now the sections of 7 µm were prepared on egg albumin coated slides.

Paraffin-embedded tissues sections in slides were, dewaxed with xylene for 10 min and rehydrated with grades of absolute alcohol, followed by 90%, 70% of isopropyl alcohol for 5 min each and then by distilled water. After dewaxing and rehydration, section blocking was done with 3% BSA (Sigma) in PBS for 1 hour in a humid chamber at room temp. Now the sections were incubated overnight at 4°C with anti-Nef antibody raised in goat (Santa cruz) using dilution of 1∶100 along with E-cadherin antibody (Santa cruz) using dilution of 1∶100 (conjugated with Alexa flour 647) in 1% BSA in PBS. Then the slides containing the sections were washed thrice with PBST and twice with PBS for 5 minutes. Now the secondary antibody of anti-goat conjugated to FITC at 1∶250 dilution in 1% BSA was added to the section and incubated further for 4 h in the dark at room temperature followed by three washings with PBST, plus two washings with PBS for 5 minutes each. The slide containing the sections were incubated with DAPI (Sigma Aldrich USA 1 µg/ml) for 20 min at RT following three washings with PBS. Finally, the slides were mounted with permanent mounting and sealing reagent Prolong gold (Invitrogen P36934) and examined through fluorescence microscopy.

### Ethics Statement

The CPCSEA/IAEC (Registration No.: 34/1999 dated 11-3-99) guide lines were strictly followed during the entire course of study. SD Rats used in this study were approved by the institutional ethical clearance committee IAEC, Approval Number: IAEC/2010/26, Central Drug Research Institute, Lucknow, UP, India.

## Supporting Information

Figure S1 **Specificity of Nef antibodies was confirmed in the parallel immuno-staining experiment as defined in** **Figure 6** **, while using the secondary antibody directly without probing with the Nef specific antibody.** The panel consists of set of figures: uterus, placenta, amniotic membrane and embryo. Blue color shows the nuclear staining with DAPI whereas overlapping images are merged with the phase pictures. The fetal tissues were isolated from rats after an hour of intra-venous injection with 500 µg of recombinant Nef protein.
(TIF)

Figure S2 **Specificity of Nef antibodies detection was confirmed in the parallel immuno-staining experiment as defined in** **Figure 6** **, while using the similar tissue sections from the negative control set of animals where the dye was injected (within an hour) alone without Nef.** The panel consist of set of figures: uterus, placenta, amniotic membrane and embryo. Blue colour shows the nuclear staining with DAPI whereas overlapping images are merged with the phase pictures.
(TIF)

Figure S3 **Standard graph of purified recombinant Nef protein in the range of 0.25 to 25 ng was made to calculate the concentration of recombinant Nef protein persists in fetal tissue lysates.**
(TIF)

Table S1 **Amount of Nef detected in plasma and migrated in the fetal organs, within an hour of HIV-1 Nef injection.**
(DOC)
