# Supplementary figures and images for: HIV-1 Nef Breaches Placental Barrier in Rat Model
Source: PLoS One. 2012 Dec 11;7(12):e51518. doi: 10.1371/journal.pone.0051518 (PMC3519864; doi:10.1371/journal.pone.0051518)

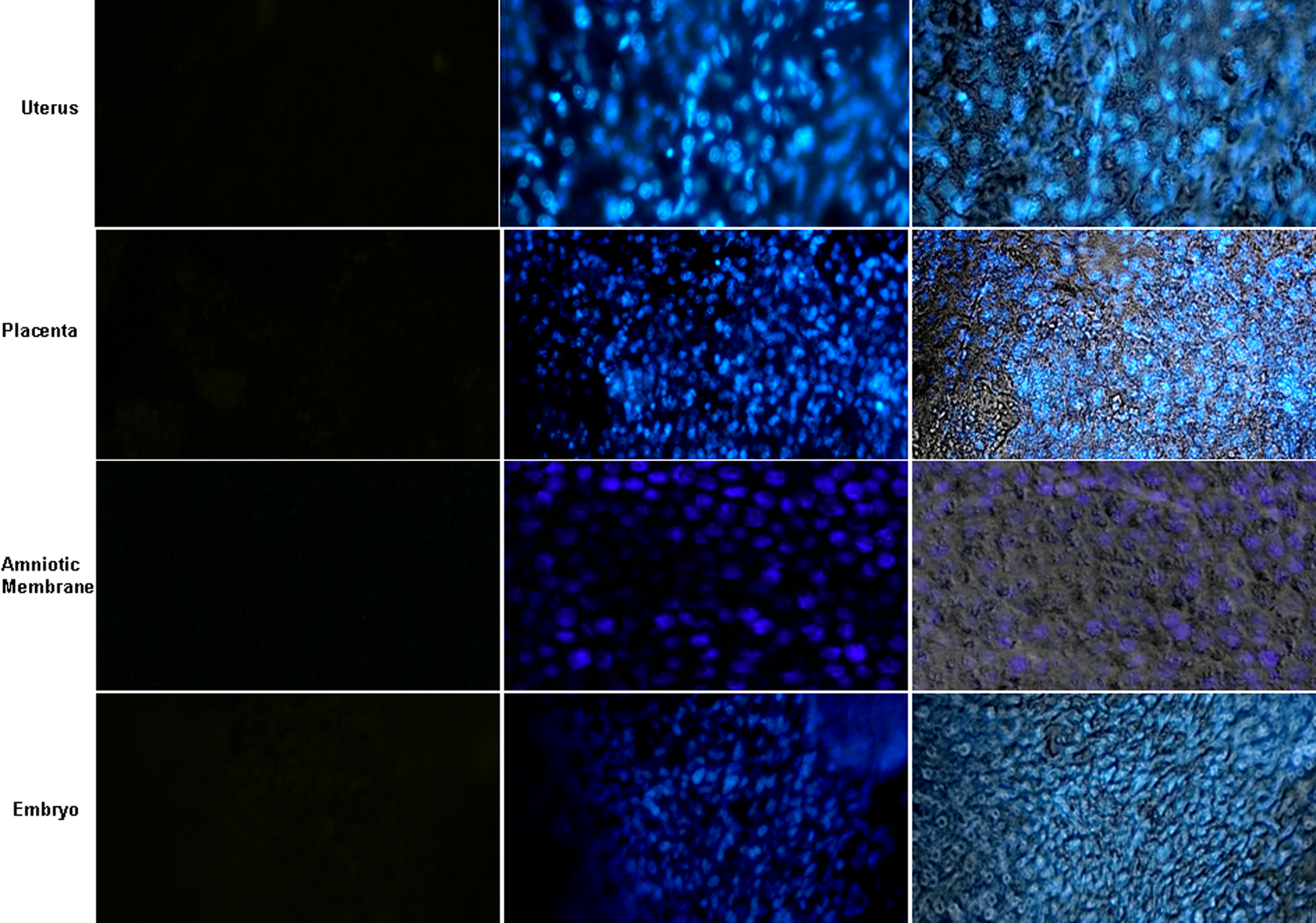

Supplement: Figure S1 — Specificity of Nef antibodies was confirmed in the parallel immuno-staining experiment as defined in Figure 6 , while using the secondary antibody directly without probing with the Nef specific antibody. The panel consists of set of figures: uterus, placenta, amniotic membrane and embryo. Blue color shows the nuclear staining with DAPI whereas overlapping images are merged with the phase pictures. The fetal tissues were isolated from rats after an hour of intra-venous injection with 500 µg of recombinant Nef protein. (TIF) [file pone.0051518.s001.tif]

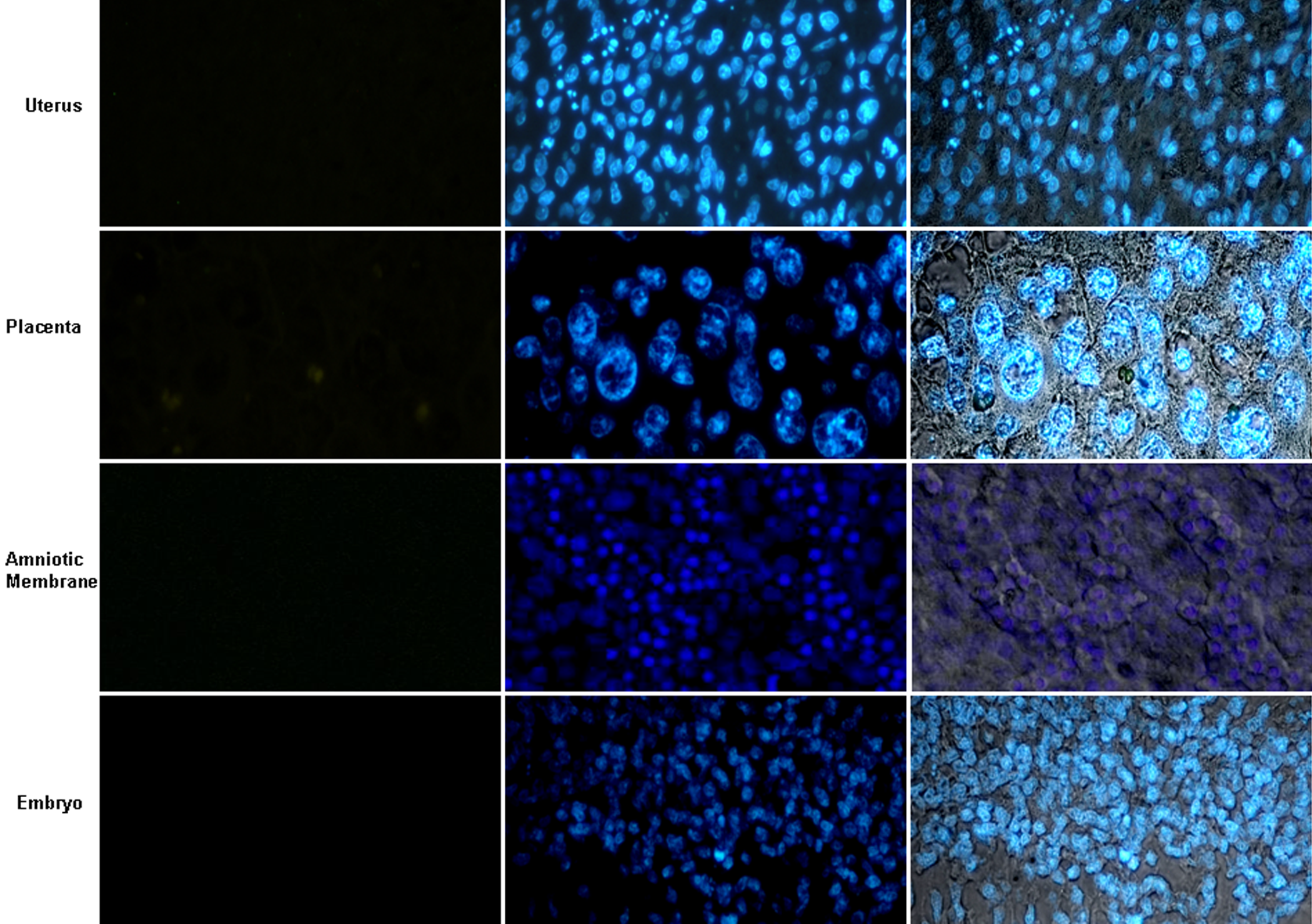

Supplement: Figure S2 — Specificity of Nef antibodies detection was confirmed in the parallel immuno-staining experiment as defined in Figure 6 , while using the similar tissue sections from the negative control set of animals where the dye was injected (within an hour) alone without Nef. The panel consist of set of figures: uterus, placenta, amniotic membrane and embryo. Blue colour shows the nuclear staining with DAPI whereas overlapping images are merged with the phase pictures. (TIF) [file pone.0051518.s002.tif]

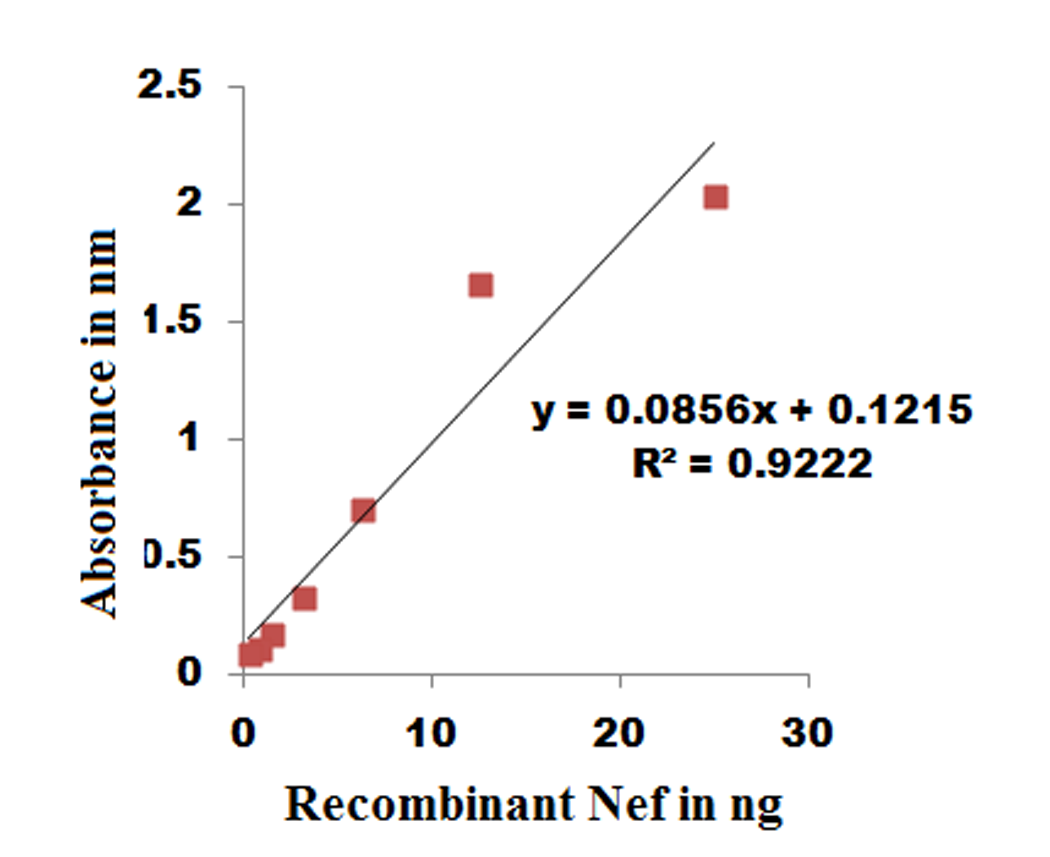

Supplement: Figure S3 — Standard graph of purified recombinant Nef protein in the range of 0.25 to 25 ng was made to calculate the concentration of recombinant Nef protein persists in fetal tissue lysates. (TIF) [file pone.0051518.s003.tif]
