# Supplementary material for: HIV-1 Nef Breaches Placental Barrier in Rat Model
Source: PLoS One. 2012 Dec 11;7(12):e51518. doi: 10.1371/journal.pone.0051518 (PMC3519864; doi:10.1371/journal.pone.0051518)
Supplement: Table S1 — Amount of Nef detected in plasma and migrated in the fetal organs, within an hour of HIV-1 Nef injection. (DOC) [file pone.0051518.s004.doc]

**Supplementary Table 1:** Amount of Nef detected in plasma and migrated in the fetal organs, within an hour of HIV-1 Nef injection.

| **Nef injected**  **(µg)** | **Conc. of Nef in Plasma(ng/ml)** | **SD (+/-)** | **Nef Migrated in Different Fetal Organs (ng/ml)** | | | | | **Effect of Nef on BBB** | **Effect of Nef on PB** |
| --- | --- | --- | --- | --- | --- | --- | --- | --- | --- |
|  |  |  | **Uterus** | **Placenta** | **Amniotic membrane** | **Amniotic fluid** | **Embryo** |  |  |
| **500** | 865 | 5.020 | 29.7 | 24.5 | 13 | 8.5 | 7.5 | Breach | Breach |
| **250** | 584 | 7.071 | 13.9 | 10.9 | 6 | 5.8 | 4.8 | Breach | Breach |
| **200** | 500 | 0.495 | 7.8 | 4.6 | 0 | 0 | 0 | Breach | Breach |
| **150** | 346 | 3.536 | 0 | 0 | 0 | 0 | 0 | No | No |
| **100** | 264 | 1.414 | 0 | 0 | 0 | 0 | 0 | No | No |
| **50** | 97 | 4.704 | 0 | 0 | 0 | 0 | 0 | No | No |
| **25** | 10 | 0.07 | 0 | 0 | 0 | 0 | 0 | No | No |
